# Resource profiles and suicide attempts in youth with disabilities

**DOI:** 10.1111/jcpp.70122

**Published:** 2026-01-30

**Authors:** Minhae Cho, C. Hyung Keun Park, Misa Kayama, Sujin Seo, Jungjoon Ihm

**Affiliations:** ^1^ School of Social Work University of Memphis Memphis TN USA; ^2^ Department of Psychiatry Asan Medical Center Seoul Republic of Korea; ^3^ Department of Social Work University of Mississippi University MS USA; ^4^ Department of Public Health Science, Graduate School of Public Health Seoul National University Seoul Republic of Korea; ^5^ Dental Research Institute, School of Dentistry Seoul National University Seoul South Korea

**Keywords:** Suicide attempt, resource profiles, youth with disabilities, early adulthood outcome

## Abstract

**Background:**

The issue of suicide among youth with disabilities transitioning into adulthood is a serious public health issue. In navigating this transition, youth with disabilities encounter unique obstacles that require careful consideration and support. This study aims to identify resource profiles among youth with disabilities and their association with suicide attempts in early adulthood.

**Methods:**

Using data from the National Longitudinal Study of Adolescent Health (Add Health), this study included 1,472 youth with disabilities. A Gaussian finite mixture model (GMM) was employed to identify underlying distinct groups of youth with disabilities based on their available resources.

**Results:**

Four latent classes emerged: *(1) Socioeconomically Advantaged and Socially Supportive (37%); (2) Socioeconomically Advantaged, but Socially Isolated (28%); (3) Socioeconomically Disadvantaged and Socially Isolated (20%); and (4) Socioeconomically Disadvantaged, but Socially Supportive (15%).* Results from the generalized linear mixed model (GLMM) considering a longer transition period into adulthood up to age 32 and relevant time‐varying factors found that youth in *Socioeconomically Advantaged, but Socially Isolated* and *Socioeconomically Disadvantaged, but Socially Supportive* had a significantly lower likelihood of suicide attempts compared to those in *Socioeconomically Advantaged and Socially Supportive.* The likelihood of suicide attempts for youth with learning disabilities was significantly lower than for those with physical disabilities, while a history of suicide attempts in adolescence and experience with a death by suicide of family members or friends increased the odds of suicide attempts.

**Conclusions:**

The study highlights the heterogeneity of youth with disabilities, demonstrating how demographic characteristics, disability‐specific needs, family and school environments and social support systems intersect to influence suicide attempt prevention.

## Introduction

Suicide among youth with disabilities is a serious public health issue as they transition into adulthood, which can increase their vulnerability to mental health issues (Cook et al., 2024). Indeed, becoming an adult presents all youth with a myriad of challenges marked by significant physical, psychological and social changes as they strive for identity development and explore newfound roles independence, and relationships (Arnett, 2007). For those with disabilities, such changes and challenges are even more profound as they take on increased responsibilities across various aspects of their lives, including accessing disability‐related services and support they need for everyday activities (e.g. Gauthier‐Boudreault, Beaudoin, Gallagher, & Couture, 2019). In navigating the transition to adulthood, youth with disabilities encounter unique obstacles that require careful consideration and support, unlike during their early childhood and school years when they were under the protection of their parents and educators, for example, through structured, formal services implemented under laws like the Individuals with Disabilities Education Act in the U.S. (IDEA; U.S. Department of Education, 2025). The IDEA, for instance, mandates that educators identify children with disabilities and offer services appropriate for their educational success (U.S. Department of Education, 2025; National Center for Learning Disabilities, 2024). The IDEA also ensures that children receive support for their transition to adulthood by age 16 or earlier, which, however, focuses primarily on academic and vocational skills (Davis & Lee, 2023; Wilt, Hirano, & Morningstar, 2021) with less attention to their mental health. Yet as youth with disabilities transition into adulthood, they lose such legal and parental protections by ‘aging out’ of formal systems. Seeking and accessing adult health care also increases the burden of youth because they need to advocate for themselves (Davis & Lee, 2023). Thus, they can benefit from continuing and consistent guidance into new services, including by family members and community practitioners (King, Baldwin, Currie, & Evans, 2005; Pandey & Agarwal, 2013; Stewart, Law, Rosenbaum, & Willms, 2001). Without family and community resources, however, many youths with disabilities struggle with this huge transition, thus rendering them especially vulnerable to mental health issues (Amalfi et al., 2023; Honey et al., 2011), which include both internalizing (e.g. anxiety, depression, withdrawal) and externalizing (e.g. aggression, impulsivity, defiance) behaviour problems (Haft, Chen, LeBlanc, Tencza, & Hoeft, 2019; O'Rourke, Brown, Martin Ginis, & Arbor‐Nicitopoulos, 2022).

Furthermore, the literature specifically highlights variations in mental health issues across disability types. Youth with disabilities involve a diverse group of adolescents, from those with physical impairments or chronic illnesses to those with intellectual, developmental, learning or sensory disabilities. A considerable body of research indicates that youth with disabilities face significantly higher rates of mental health issues, including anxiety, depression, attention‐deficit hyperactivity disorder and emotional or behavioural disorders. However, the susceptibility to specific mental health challenges can differ depending on the type of disability, as each disability can present unique emotional and psychological vulnerabilities, influenced by the intersection of biological, psychological and social factors related to the condition (Casey Foundation, 2024; Danielsson et al., 2024; Skaar, Etscheidt, & Kraayenbrink, 2021; Totsika, Liew, Absoud, Adnams, & Emerson, 2022). For example, youth with comorbid and developmental disabilities, including intellectual disabilities, tend to have more psychiatric problems with peers than those with physical disabilities (O'Rourke et al., 2022). Moreover, youth with intellectual and developmental disabilities (e.g. Down syndrome, autism, fetal alcohol syndrome) run a greater risk of anxiety, depression and psychotic illness when compared to their typically developing peers (King et al., 2019; Marquis et al., 2024). Furthermore, as youth with intellectual and developmental disabilities age from 15 to 24 years old and experience the loss of structured daily activities and limited employment opportunities, the prevalence of anxiety and depression also increases (Marquis et al., 2024; Hudson, 2006).

School‐aged youth with specific learning disabilities and ADHD also tend to struggle with interpersonal relationships and academic skills, resulting in greater depressive symptoms and interpersonal conflicts than typically developing youth (Haft et al., 2019). Similarly, youth (14–25 years) with physical disabilities reported mental health issues, such as somatization, depression, low self‐esteem and feelings of inadequacy, especially among those who reported greater functional limitations due to disabilities that restrict their social participation (Amalfi et al., 2023). Stigma associated with disabilities and a lack of accessible community resources and activities also can lead to lower social participation among youth with physical disabilities relative to their typically developing peers (Tanure Alves, Grenier, Haegele, & Duarte, 2020). This may, in turn, lead to social isolation and decreased quality of life, potentially rendering youth more vulnerable to mental health issues, including suicidality, defined as encompassing suicidal ideation, suicidal behaviour and death by suicide, commonly referred as ‘suicide’ (Cervantes et al., 2022).

While suicide and suicidality among youth have been seriously addressed in general, those among youth with disabilities have received significantly less attention even though factors related to disability (e.g. chronic pain, social isolation, stigma, bullying, limited access to healthcare) can influence suicidality (Lund et al., 2020; Merrick et al., 2006). During adulthood, high rates of co‐occurring disability, mental health issues, and unemployment can increase the risk of suicidality (Lund, Nadorff, Thomas, & Galbraith, 2020). Likewise, having children with disabilities negatively affects the socioeconomic status of their family, which can delay the access of children to appropriate services (e.g. Donley, King, Nyathi, Okafor, & Mbizo, 2018) and may increase their suicidality.

Indeed, although the prevalence of suicide among youth with disabilities is somewhat inconsistent (King et al., 2019; Lunsky, Raina, & Burge, 2012), recent studies have consistently found that youth with disabilities are more likely than the general population to experience suicidality (Cassidy et al., 2018; Cervantes et al., 2022). Using the U.S. National Emergency Department Sample databases, Cervantes et al. (2022) found statistically significant differences in the prevalence of emergency department visits for suicidal ideation or intentional self‐inflicted injury in youth with autism (5.1%) or intellectual disabilities (ID) (6.6%), compared to youth without disability (1.2%). Additionally, Cervantes et al. (2022) found that the highest rates of emergency department visits for suicidality were observed among youth aged 19–25 with ID (8.3%) and autism (7.4%), in contrast to 1.2% in the comparison group, followed by ages 13–18 where the autism group had the highest rate (7.2%), and ages 7–12, which also showed elevated rates among those with autism (2.0%) and ID (1.3%), in contrast to the comparison group (0.3%). Co‐occurring mental health conditions also placed youth with disabilities at higher risk for self‐harm (OR: 2.54), suicidal ideation (OR: 2.54) and attempted suicide (OR: 3.48). Given the high prevalence of suicidality among youth with disabilities, identifying distinct subgroups with varying levels of risk is crucial for designing effective prevention programmes. However, little research focuses specifically on youth with disabilities.

Previous research has collectively identified various resources as protective factors for developmental outcomes of youth with disabilities, such as family and community‐based support (King et al., 2005; Pandey & Agarwal, 2013). However, much existing research has employed variable‐centred approaches to model associations between the presence or absence of those factors and their developmental outcomes. Although such approaches help us understand the relative contribution of a specific factor to a group outcome, they obscure underlying characteristics of youth with disabilities, particularly characteristics related to their available resources, which may lead to variations in their adult outcomes. Building on the literature, this study aimed to identify heterogeneity in available resources among youth with disabilities and to investigate its association with suicide attempts in early adulthood. Three research questions guided this study: (1) What resource profiles can be found for youth with disabilities? (2) To what extent do demographic characteristics differ across identified resource profiles? and (3) How are resource profiles among youth with disabilities associated with suicide attempts in early adulthood?

## Methods

### Data and sample

The U.S. National Longitudinal Study of Adolescent Health (Add Health) provides a comprehensive longitudinal data set detailing the health‐related behaviour of a diverse cohort of adolescents in grades 7 through 12. Conducted during the 1994–1995 academic year, Wave 1 surveyed 90,118 adolescents, focusing on adolescent health determinants and associated risk behaviours. Spanning April to August 1996, Wave 2 involved in‐home interviews with 14,738 youths aged 12–21, all of whom participated in Wave 1. From 2001 to 2002, Wave 3 gathered data from 15,197 emerging adults aged 18–28, concentrating on their decisions and behaviours as they transitioned into adulthood. Conducted from 2008 to 2009, Wave 4 included 15,701 respondents aged 24–32. Collectively, Waves 1–4 span approximately 13 years of development, tracking youth from adolescence to early adulthood. In this study, we used Wave 1 data collected from 1,472 youth with disabilities aged approximately 12–18 years old (7th–12th grade) in the 1994–1995 academic year as baseline data and followed them through Waves III and IV on their suicide attempts and other variables during early adulthood.

### Measures

#### Suicide attempts

Self‐reports of suicide attempts were collected at Wave III (ages 18–26) and Wave IV (ages 24–32). Respondents indicated how many times they had attempted suicide in the past 12 months, with responses categorized as ‘none’, ‘once’, ‘twice’, ‘3–4 times’ and ‘5 or more times.’ For analysis, suicide attempt was recoded as a binary variable: 0 for none and 1 for any attempt.

#### Family socioeconomic status

In Wave I, parents reported their socioeconomic status. *Financial stability* was created as a count variable ranging from 0 to 6. Parents were asked to indicate their status (yes/no) in any of the following six welfare receipts: (1) Social Security or Railroad Retirement, (2) Supplemental Security Income (SSI), (3) Aid of Families with Dependent Children (AFDC), (4) food stamps, (5) unemployment or workers' compensation and (6) a housing subsidy or public housing. After summing the number of welfare receipts, the values were reverse‐coded, so a higher score indicated a better financial status. *Annual household income* was categorized into quintiles based on 1994 U.S. Census data (U.S. Bureau of the Census, 1996). *Parental occupation* was classified into six levels, ranging from 0 for unemployment to 5 for professions such as doctor, lawyer, scientist, etc. *Parental education* was measured by the highest educational level obtained by either parent on a 5‐level ordinal variable, ranging from 1 for less than high school to 5 for professional training beyond college.

### Social supports

This study utilized the In‐Home Questionnaire at Wave I for six constructs of social supports. First, *religiosity* was assessed using four items including religious affiliation, religious service attendance, religious salience and scripture beliefs. To be specific, religious affiliation was measured as a binary variable: 0 for non‐religious and 1 for any of the eight religious traditions based on the RELTRAD method (Steensland et al., 2000). Religious service attendance was measured as an ordinal variable, ranging from 0 for never attending to 4 for attending weekly or more. Religious salience was measured using a 4‐point Likert scale, ranging from 1 for *not important at all* to 4 for *very important*. Lastly, scripture beliefs were measured as a binary variable through one question regarding the agreement of the respondent with the sacred scriptures as the word of God: 0 for those who disagree or whose religion does not have sacred scriptures and 1 for those who agree. Second, *social cohesion* was measured by three indicators including interaction with neighbours in the neighbourhood, mutual concern and support among residents in the neighbourhood and familiarity with most people residing in the neighbourhood. Third, *family relationships* were assessed through five criteria such as perceived parental care, understanding within the family, the desire to leave home, engaging in fun activities as a family and receiving attention from the family. Fourth, to measure *peer relationships*, four elements were considered: the care from friends, perceived prejudices among school peers, rapport with fellow students (reverse coded) and a sense of social acceptance (reverse coded). The reverse coding ensured that higher scores indicated stronger peer bonds. Fifth, teacher relationships were assessed through three elements: perceived care from teachers, rapport with teachers (reverse coded) and the perception of fairness in teacher–student interactions (reverse coded). Higher scores indicated more positive bonds with teachers. Sixth, school attachment was determined by how close the respondents felt to schoolmates, a sense of belonging within the school, satisfaction with being at the school and the feeling of safety in the school environment. After reverse coding, higher scores reflected a stronger sense of school belonging.

#### Covariates

A set of variables was included as covariates, including Wave I sociodemographic information during adolescence and early adulthood. *Sex* was dichotomously categorized with males coded as 0 and females as 1. *Race* was treated as a categorical variable, encompassing White, Black or African American and Other (e.g. Hispanic and Asian). To examine potential variations in suicide attempts by disability type, this study identified specific types of disability based on the availability of data in the Add Health data set. Add Health provides data for three distinct disability types: physical, intellectual and learning (Chen & Harris, 2020). *Disability types* included physical, learning, intellectual and multiple disabilities. The identification of *physical disabilities* was based on youth self‐report of functional limitations or the need for mobility aids (e.g. walking aid device, brace on arms or leg or artificial limb). *Learning disabilities* were recognized if a parent affirmed a specific learning disorder and noted the adolescent had used special education services during the preceding year. *Intellectual disabilities* were recognized based on parental acknowledgment of cognitive impairment, paired with scores from the Add Health Picture Vocabulary Test (AHPVT), a condensed variant of the revised Peabody Picture Vocabulary Test, which correlates with intelligence tests like the Wechsler Intelligence Scale for Children (Harris & Halpern, 2022). Adolescents were considered intellectually disabled if their parents identified them as such or if they scored below 75 on the AHPVT. During the administration of the Add Health survey, trained data collectors assisted adolescents who might have faced challenges in completing the survey independently to ensure accurate data collection from participants (Harris, 2013). For respondents who indicated more than one specified type of disability, this study identified them as having multiple disabilities.

Considering pre‐existing risk factors, the study included *suicide attempts during adolescence* as a binary variable, measured at Wave I and/or Wave II when the participants were in grades 7–12: 0 for none and 1 for any attempt. From youth self‐reports at Wave III and Wave IV, the study also included four time‐varying measures. *Independent living* was assessed as a binary variable through youth self‐report. *Depressive symptoms* were measured using a modified 9‐item version of the Center for Epidemiologic Studies Depression Scale (CES‐D‐9). The CES‐D‐9 demonstrates good internal consistency, with Cronbach's alpha coefficients ranging from 0.8 to 0.9 (Lewinsohn et al., 2003; Kim & Hwang, 2016). Although the instrument has not been specifically tailored for youth with disabilities, its psychometric properties are generally considered acceptable for use in various youth populations, including those with chronic health conditions (Fountoulakis et al., 2001) or ID (Olivier et al., 2022). The response options included: never or rarely = 0, *sometimes* = 1, *a lot of the time* = 2 and *most of the time or all of the time* = 3. Positive experiences such as ‘You enjoyed life’ were reverse coded. These responses were converted to binary variables, with 1 indicating *depressive symptoms*, including a *lot of the time* = 2 and *most of the time or all of the time* = 3, and 0 indicating *no depressive symptoms*, including *never or rarely* = 0 and *sometimes* = 1. Then, a mean score was calculated, with higher scores indicating more depressive symptoms. *Experience of a death by suicide among family members or friends* was assessed using a binary measure: 1 if the respondent indicated any death occurred due to suicide, either involving a close family member or a friend, and 0 otherwise. *Individual income* for youth was reported by youth and measured as a continuous variable in Waves III and IV, respectively.

### Analytic plan

Descriptive statistics were conducted to describe the characteristics of the entire sample and across the identified classes. A Gaussian finite mixture model (GMM) was employed to identify underlying distinct groups of youth with disabilities based on their available resources. The GMM iteratively estimates the parameters of the underlying distributions, including their means and variances, adjusting the parameters to maximize the likelihood of observing the actual data. Once the model was fitted, individuals were assigned to the mutually exclusive clusters that best represented them, based on their observed characteristics related to resources. Clustering variables included financial stability, parent income, occupation, education, religiosity, social cohesion, family and peer relations, school attachment and teacher relations. The optimal number of clusters was determined based on a combination of Bayesian information criterion (BIC), integrated completed likelihood (ICL) and likelihood ratio test (LRT) values, along with a qualitative interpretation of the identified clusters. The model, fitted using the EM algorithm, employed the VVV model, which assumes ellipsoidal clusters with variable volume, shape and orientation. Cluster interpretation was aided by a bootstrap procedure, scaling the mean parameters for comparability. Analyses were conducted in R (version 4.3.0) using the ‘mclust’ package (Scrucca, Fop, Murphy, & Raftery, 2016), with visualizations generated in ggplot2.

## Results

### Sample characteristics and descriptive statistics for key variables

Table 1 presents demographic characteristics and descriptive statistics for our sample. Following the Add Health guidelines, each individual was weighted longitudinally as Wave I respondents were interviewed at Wave III and Wave IV (Chantala & Suchindran, 2011). In Add Health Wave I restricted data set, the total weighted sample comprised 21.1% of adolescents with disabilities, including 3.2% with physical disabilities, 2.4% with intellectual disabilities, 11% with learning disabilities and 3.5% with multiple disabilities. The sex composition among study participants included 55% males. The sample mean of financial stability was 5 (*SD* = 1). Average annual household income was reported as 3 (*SD* = 1). The mean level of parental occupation was 2 (*SD* = 2), and for the parent education level, 3 (*SD* = 1). For social support, the religiosity mean was 5 (*SD* = 3), and for social cohesion, it was 2 (*SD* = 1). The average score for family relationships was 18 (*SD* = 3), whereas for peer relationships, it was 16 (*SD* = 2). Similarly, teacher relationships had a mean of 12 (*SD* = 2), and the school attachment mean was 15 (*SD* = 3). For time‐varying variables, during Wave 3, when the youths were aged 18–26, only 7.5% lived independently, with a slight increase to 9.5% at Wave 4 (ages 24–32). As for loss of family members or friends by suicide, 38% of youths experienced this in Wave 3 and 7.3% in Wave 4. The mean individual income for youth was $11,596 (*SD* = 13,703) in Wave 3 and $32,158 (*SD* = 37,484) in Wave 4. The mean score of depressive symptoms was 0.7 (*SD* = 0.39) at Wave III and 0.8 (*SD* = 0.4) at Wave III. In Wave 3, 1.8% of youths reported suicide attempts, increasing slightly to 2% in Wave 4. Overall, 4.2% reported suicide attempts in either Wave 3 or Wave 4.

**Table 1:** Weighted sample descriptive statistics (*N* = 1,472)

Variables^a^	*N* (%)
Type	
Physical	368 (25%)
Intellectual	113 (7.7%)
Learning	846 (57%)
Multiple	145 (9.9%)
Sex	
Female	666 (45%)
Male	806 (55%)
Race	
White	927 (63%)
Black or African	256 (17%)
Other	289 (20%)
Family socioeconomic status (Mean, *SD*)	
Financial stability	5 (1)
Annual household income	3 (1)
Parental occupation	2 (2)
Parent education	3 (1)
Social support (Mean, *SD*)	
Religiosity	5 (3)
Social cohesion	2 (1)
Family relationships	18 (3)
Peer relationships	16 (2)
Teacher relationships	12 (2)
School attachment	15 (3)
Suicide attempt during adolescence	
No	1,429 (97.1%)
Yes	43 (2.9%)
Time‐varying variables	
Independent living	
*Wave3* Alone	110 (7.5%)
w/others	1,309 (89%)
*Wave4* Alone	139 (9.4%)
w/others	1,315 (89%)
Family/friend suicide	
*Wave3* No	1,273 (86%)
Yes	554 (37.6%)
*Wave4* No	1,352 (92%)
Yes	107 (7.3%)
Youth individual income	
*Wave3* Mean (*SD*)	11,596 (13,703)
*Wave4* Mean (*SD*)	32,158 (37484%)
Depressive symptoms	
*Wave3* Mean (*SD*)	0.70 (0.39)
*Wave4* Mean (*SD*)	0.79 (0.40)
Outcome variables	
Suicidality attempt	
No	1,418 (96.3%)
Yes	54 (3.7%)
*Wave3* No	1,445 (98.2%)
Yes	27 (1.8%)
*Wave4* No	1,442 (98%)
Yes	30 (2%)

a Counts are presented as unweighted counts, and percentage, mean and *SD* are weighted.

### Interpretation of the four‐class model of youth with disabilities

**Figure 1:**
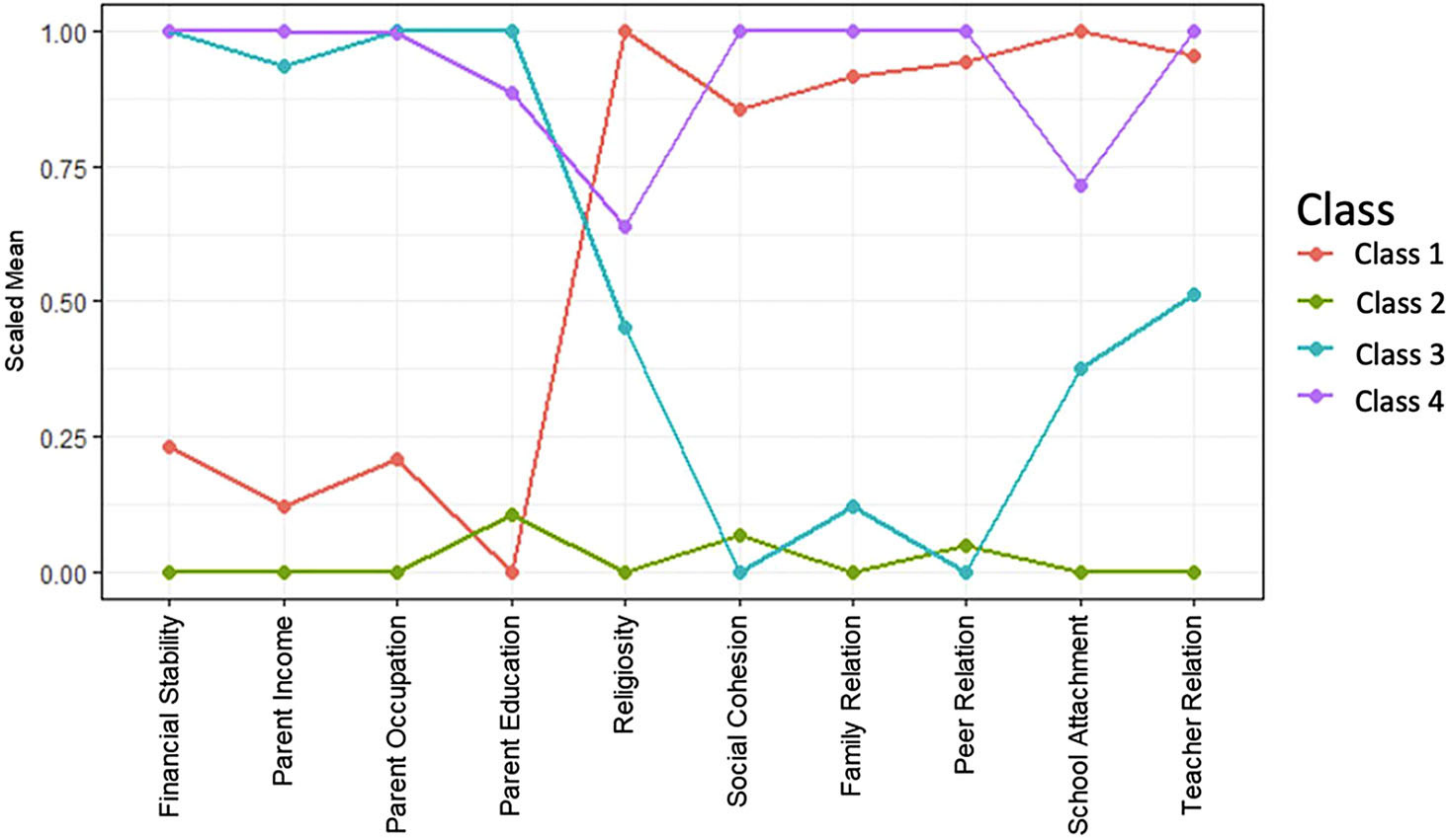
Scaled mean of variables for the four‐class Gaussian mixture model. Class 1: Socioeconomically disadvantaged, but socially supportive; Class 2: Socioeconomically disadvantaged and socially isolated; Class 3: Socioeconomically advantaged, but socially isolated; and Class 4: socioeconomically advantaged and socially supportive

After a comprehensive consideration of the available resources of participants, as well as both parsimony and complexity, we determined that a four‐class model was optimal (see Appendix S1). Fit indices, such as AIC, BIC and ICL, for each group number were calculated, as displayed in Table S1 and Figure S1 visualizes the comparisons between the three models: two, three and five groups. Figure 1 illustrates the four‐class model of available resources among youth with disabilities: 15% were classified into Class 1; 20% into Class 2; 28% into Class 3; and 37% into Class 4. Class 1, the smallest group, labelled *Socioeconomically Disadvantaged but Socially Supportive*, consisted of youth with the highest probability of coming from socioeconomically disadvantaged families but also becoming psychosocially resilient. More specifically, this group exhibited low levels in family sociodemographic characteristics (i.e. financial stability, income, occupation and education) but compensated with high levels of psychosocial factors (i.e. religiosity, social cohesion, family relationships, peer relationships, school attachment and teacher relationships), which may act as protective factors despite economic challenges. Class 2, the second smallest, labelled *Socioeconomically Disadvantaged and Socially Isolated*, represented youth with low levels in all variables, indicating a vulnerable socioeconomic, psychosocial profile. These youth face challenges in family stability, income, parent occupation and parent education, along with lower levels of religiosity, social cohesion, family relationships, peer relationships, school attachment and teacher relationships. Class 3, labelled *Socioeconomically Advantaged, but Socially Isolated*, was characterized by high levels of family socioeconomic characteristics but low levels of psychosocial factors. Despite their advantageous socioeconomic backgrounds, these youth with disabilities likely faced challenges in religiosity, social cohesion, family relationships, peer relationships, school attachment and teacher relationships. Class 4, the largest group, labelled *Socioeconomically Advantaged and Socially Supportive*, was characterized by high levels of family financial stability, household income, parent occupation and parent educational level, suggesting a strong socioeconomic background. These youth exhibited high levels of social support from multilevel systems.

### Assessment of sociodemographic invariance across latent profiles

Analysing the demographic characteristics by group using the chi‐squared test (see Table 2), we found significant associations between class and disability type (*p* < .001). Physical disabilities varied across classes, with the proportion highest in Class 3 (27%). Intellectual disabilities were the most prevalent in Class 1 (10%) and Class 2 (9.6%), and less common in Class 3 (5.7%) and Class 4 (3.8%). Classes 1 and 2 also had higher proportions of youth identifying as Black or African American, with 29% in Class 1 and 27% in Class 2 compared to 7.8% in Class 3 and 9.7% in Class 4. Learning disabilities consistently represented the highest proportions across all classes, ranging from 50% to 66%. Multiple disabilities were particularly elevated in Class 2 (23%). No significant sex differences were observed across classes. Racial composition differed significantly (*p* < .001), with White youth being predominant but varying from 52% in Class 1 to 78% in Class 4. Family socioeconomic status variables also showed significant differences across classes (*p* < .001). Regarding social support, significant differences were found in religiosity, social cohesion, peer relationships and school attachment (*p* < .001). Suicide attempts during adolescence, measured at Wave I and/or Wave II when the participants were in grades 7–12, differed significantly by class (*p* < .05), but no differences were observed in independent living, family/friend suicide history or depressive symptoms. Individual income increased from Wave 3 to Wave 4, with significant differences between classes in Wave 4 (*p* < .001).

**Table 2:** Sample descriptive statistics across latent classes

Variables^a^	Class 1	Class 2	Class 3	Class 4	*p*‐Value^b^
	*N* = 214 (15%)	*N* = 272 (20%)	*N* = 459 (28%)	*N* = 527 (37%)	
Type					
Physical	53 (17%)	55 (17%)	129 (27%)	131 (22%)	**<0.001**
Intellectual	23 (10%)	25 (9.6%)	38 (5.7%)	27 (3.8%)	
Learning	119 (66%)	142 (50%)	255 (58%)	330 (66%)	
Multiple	19 (6.6%)	50 (23%)	37 (9.3%)	39 (8.8%)	
Sex					
Female	97 (39%)	134 (39%)	202 (39%)	233 (40%)	0.988
Male	117 (61%)	138 (61%)	257 (61%)	294 (60%)	
Race					
White	101 (52%)	142 (57%)	307 (77%)	377 (78%)	**<0.001**
Black or African	67 (29%)	74 (27%)	49 (7.8%)	66 (9.7%)	
Other	46 (19%)	56 (16%)	103 (15%)	84 (12%)	
Family socioeconomic status					
Financial stability	4 (1)	4 (1)	6 (0)	6 (0)	**<0.001**
Annual Household income	2 (1)	2 (1)	3 (1)	3 (1)	**<0.001**
Parental occupation	2 (2)	2 (2)	3 (2)	3 (2)	**<0.001**
Parent education	2 (1)	2 (1)	3 (1)	3 (1)	**<0.001**
Social support					
Religiosity	6 (2)	3 (3)	5 (3)	5 (3)	**<0.001**
Social cohesion	3 (0)	2 (2)	1 (1)	3 (0)	**<0.001**
Family relationships	19 (3)	18 (3)	18 (2)	18 (2)	0.605
Peer relationships	16 (2)	16 (2)	16 (2)	17 (2)	**<0.001**
Teacher relationships	12 (2)	12 (3)	12 (2)	12 (2)	0.12
School attachment	16 (2)	13 (4)	14 (3)	15 (3)	**<0.001**
Suicide attempt at Wave II					
No	204 (95.3%)	364 (97.1%)	442 (96.3%)	519 (98.5%)	**0.010**
Yes	10 (4.7%)	8 (2.9%)	17 (3.7%)	8 (1.5%)	
Time‐varying variables					
Independent living					
*Wave3* Alone	12 (5.3%)	21 (7.3%)	36 (8.7%)	41 (9.5%)	0.465
w/others	199 (95%)	243 (93%)	410 (91%)	457 (90%)	
*Wave4* Alone	19 (8.9%)	21 (7.6%)	48 (11%)	51 (10%)	0.646
w/others	192 (91%)	245 (92%)	407 (89%)	471 (90%)	
Family/friend suicide					
*Wave3* No	186 (87%)	235 (89%)	406 (91%)	446 (87%)	0.438
Yes	23 (13%)	28 (11%)	40 (8.6%)	63 (13%)	
*Wave4* No	197 (95%)	243 (89%)	426 (92%)	486 (92%)	0.420
Yes	13 (5.3%)	22 (11%)	32 (7.6%)	40 (8.0%)	
Individual income					
*Wave3* Mean (*SD*)	14,005 (14,404)	12,616 (11,766)	12,901 (12,618)	11.348 (13,828)	0.136
*Wave4* Mean (*SD*)	25,061 (20,474)	20,147 (20,883)	32,545 (28,508)	33,045 (39,108)	**<0.001**
Depressive symptoms					
*Wave3* Mean (*SD*)	0.67 (0.40)	0.74 (0.38)	0.70 (0.38)	0.69 (0.39)	0.217
*Wave4 Mean* (*SD*)	0.76 (0.40)	0.82 (0.45)	0.79 (0.37)	0.77 (0.39)	0.622
Outcome variable					
Suicidality attempt					
No	202 (96%)	263 (95%)	444 (98%)	509 (95%)	0.342
Yes	12 (4.4%)	9 (5.2%)	15 (2.2%)	18 (5.0%)	
*Wave3* No	10 (65%)	17 (84%)	28 (88%)	24 (57%)	0.060
Yes	5 (35%)	3 (16%)	8 (12%)	11 (43%)	
*Wave4* No	203 (98%)	260 (96%)	449 (99%)	517 (97%)	0.445
Yes	7 (2.4%)	6 (4.2%)	8 (1.4%)	9 (2.5%)	

Bold values indicate statistical significance (*p*‐value < 0.05).

^a^ *n* (%).

^b^ Chi‐squared test with Rao and Scott's second‐order correction and Wilcoxon rank‐sum test for survey data; Counts are presented as unweighted counts, and percent, mean, *SD* and *p*‐value are weighted.

### Suicide attempts in early adulthood across latent profiles

Given the time‐varying factors between Wave 3 and Wave 4, such as independent living and family/friend suicide history, the GLMM analysis found significant predictive validity for the latent classes (see Table 3). Youth with disabilities in Class 2 *Socioeconomically Disadvantaged and Socially Isolated* (*β* = −41.83, *p* < .004) and Class 3 *Socioeconomically Advantaged but Socially Isolated* (*β* = −20.03, *p* < .003) were less likely to attempt suicide compared to those in Class 4 *Socioeconomically Advantaged and Socially Supportive*. Youth with learning disabilities were less likely to attempt suicide (*β* = −17.20, *p* < .001) compared with those with physical disabilities. Suicide attempts during adolescence significantly increased the likelihood of suicide attempts in early adulthood (*β* = 19.68, *p* < .001). Among time‐varying factors, a strong association was noted only between family/friend suicide history and increased suicide attempts (*β* = 11.464, *p* < .001). Youth with disabilities who have experienced a death by suicide among family members or friends had an average increase of approximately 11 units in the likelihood of attempting suicide compared to those who had not experienced such a loss.

**Table 3:** Estimates of generalized linear mixed model of suicide attempt

	Wave III and Wave IV (Age 18–32)			
	Generalized linear mixed model			
	Estimate	*z*‐Value	*p*‐Value	95% CI
(Intercept)	−18.117	−1.91	.057	−36.74, 0.51
Sex: Male^a^	−1.071	−0.34	.734	−7.24, 5.10
Race^b^				
Black	−0.483	−0.08	.938	−12.58, 11.61
Other	0.192	0.05	.957	−6.72, 7.11
Class^c^				
Class 1	−0.420	−0.11	.912	−7.88, 7.04
Class 2	−41.829	−2.88	.**004**	−70.34, −13.32
Class 3	−20.026	−2.99	.**003**	−33.17, −6.88
Disability type^d^				
Intellectual	3.25	0.59	.555	−7.55, 14.05
Learning	−17.20	−4.00	**<.001**	−25.62, −8.77
Multiple	0.93	0.21	.835	−7.85, 9.71
Suicide attempts during adolescence^f^	19.68	3.59	**<.001**	8.94, 30.42
Depressive symptoms	0.07	0.02	.981	−5.68, 5.81
Independent living^e^	−1.10	−0.30	.766	−8.30, 6.11
Family/friend suicide^f^	20.53	3.79	**<.001**	9.91, 31.15
Individual income^g^	0.82	1.27	.203	−0.44, 2.08

Bold values indicate statistical significance (*p* < 0.05).

Class 1: Socioeconomically Disadvantaged, but Socially Supportive; Class 2: Socioeconomically Disadvantaged and Socially Isolated; and Class 3: Socioeconomically Advantaged, but Socially Isolated.

^a^ Reference group: Female.

^b^ Reference group: White.

^c^ Reference group (Group 4): *Socioeconomically Advantaged and Socially Supportive*.

^d^ Reference group: Physical.

^e^ Reference group: Alone.

^f^ Reference group: No.

^g^ Income variable was log‐transformed.

## Discussion

This study identified profiles that differ in socioeconomic characteristics and social support among youth with disabilities and explored the association between these profiles and suicide attempts in early adulthood. Four resource profiles (Classes 1‐4) were identified as the most appropriate. In further analysis comparing the likelihood of suicide attempts across the identified profiles, youth with disabilities in Class 2, *Socioeconomically Disadvantaged and Socially Isolated*, and Class 3, *Socioeconomically Advantaged but Socially Isolated*, were less likely to attempt suicide compared to those in Class 4, *Socioeconomically Advantaged and Socially Supportive*. Regarding disability type, the likelihood of suicide attempts was lower for youth with learning disabilities than for those with physical disabilities. Controlling for these factors, the study also found that suicide attempts during adolescence and experiencing the death of a family member or friend by suicide strongly predicted suicide attempts in early adulthood among youth with disabilities. These findings provide several meaningful discussion points.

First, variables in social supports, such as religiosity, social cohesion, family relation, peer relation, school attachment and teacher relation, all reflect the degree of social connection, with a lower level corresponding to greater social isolation. Our profile analysis result seems to contradict existing literature because it challenges the established understanding that social isolation increases the risk of suicide attempts (Motillon‐Toudic et al., 2022). The interpersonal theory of suicide (Van Orden et al., 2010), which explains the occurrence of death by suicide, suggests that when thwarted belongingness (a feeling of being alone) and perceived burdensomeness (a feeling of being burden) occur simultaneously, a desire for suicide emerges. Given the sample of youth with disabilities, however, the findings of this study may offer an alternative interpretation. For youth with disabilities, most of whom attended high school during the initial survey, socially isolated youth with disabilities may have received heightened psychological attention, including through special education (see U.S. Department of Education, 2025), such as careful monitoring and targeted interventions at school and community‐based organizations to enhance their mental health, all of which could have helped prevent psychological crisis and, eventually, suicide attempts. Worthy of note is that this finding was observed irrespective of the family socioeconomic background. Given that family socioeconomic status positively influences mental health literacy (Holman, 2014), this finding is particularly encouraging because it implies that the mental health literacy of family members might not have been a crucial factor in promoting mental health related to suicide attempts.

The findings of this study that learning disabilities serve as a protective factor against suicide attempts in early adulthood could be similarly explained. This study identified youth with learning disabilities based on reports from parents, such as the receipt of special education services by their children under the category of specific learning disorders. In the United States, eligibility to receive special education under the category of specific learning disabilities is typically determined by qualified professionals, including a school psychologist, a special education expert and a regular classroom teacher (Learning Disabilities Association of America, n.d.; U.S. Department of Education, 2025). Many also are diagnosed in elementary grades (Li et al., 2023). Thus, youth with learning disabilities and their families are likely to have engaged with mental health professionals early in life. Managing learning disabilities requires substantial time and resources. Given the increased risk of comorbid psychiatric disorders among individuals with specific learning disabilities (Espinas, Vaughn, & Fuchs, 2025; Khodeir, El‐Sady, & Mohammed, 2020), these youth likely received ongoing psychological support. Moreover, regular interactions with special education teachers and clinical professionals may have enabled parents to be more attentive to the mental health needs of their children (Jacobs et al., 2016; Semchishen & Colman, 2024). Thus, while youth with learning disabilities are vulnerable to comorbid psychiatric disorders, timely interventions may have contributed to improved mental health outcomes. Although this finding, along with that from the resource profile, appears positive and promising, further research integrating information on the psychological management of youth with disabilities from socially isolated backgrounds is needed to validate this hypothesis.

As a well‐established predictor of suicide attempt and death by suicide, history of suicide attempts during adolescence indicates increased risk of suicide attempts during early adulthood. In particular, suicide attempts during adolescence are associated with increased risk not only of subsequent suicide attempts but also of developing major depression, the most common psychiatric disorder among adolescents exhibiting suicidal behaviour, during young adulthood, ages 18–21 and 21–25 (Fergusson, Horwood, Ridder, & Beautrais, 2005). Although risk decreases somewhat later in young adulthood, it remains alarmingly high, with a risk of major depression 47.1 times greater and of suicide attempts 15.7 times greater (Fergusson et al., 2005). Youth with disabilities who have a history of suicide attempts may experience ongoing mental health challenges as they transition into adulthood. Given the heightened risk of repeated attempts or additional mental health issues, we note that these youth require routine psychiatric evaluations to assess and manage their suicide risk. Regular assessments can ensure continuity of care, facilitate a smooth transition from paediatric to adult mental health services and ultimately support their long‐term emotional well‐being and stability. Additionally, considerable literature supports familial history of suicidal behaviour increasing risks of suicide and suicide attempts among young people, which is a possible consequence of both biological and psychological processes (Alvarez‐Subiela, Castellano‐Tejedor, Villar‐Cabeza, Vila‐Grifoll, & Palao‐Vidal, 2022; Calderaro et al., 2022; Lee et al., 2022). As noted, the interpersonal theory of suicide (Van Orden et al., 2010) posits that lethal or near‐lethal suicide attempts are facilitated by the acquired capability for suicide, perhaps developing through exposure to distressing events, which subsequently increase susceptibility to suicidal behaviour by diminishing fear through habituation. For youth with disabilities who experience the suicide death of a close acquaintance or loved one, ongoing psychological monitoring is crucial to ensure timely intervention and support.

Although the resource profile measures aimed to address the complexity of family socioeconomic status and social supports by assessing multiple well‐known domains (e.g. Donley et al., 2018; King et al., 2005; Lund et al., 2020; Pandey & Agarwal, 2013; Stewart et al., 2001), it is important to note that these measures are cross‐sectional rather than longitudinal. Family socioeconomic status and availability of social resources may change as families transition through life stages. Due to data limitations providing only one time point (the youth were between the 7th and 12th grades), the analysis of the study could not examine family socioeconomic status and social resources as time‐varying variables or capture changes in resource profiles. We considered the temporal sequence between resource profiles and suicide attempts, but using time‐invariant measures for resource‐related variables may limit our ability to establish causality. Future research replicating this study with consideration of temporal changes in resource profiles could provide a more comprehensive, accurate understanding of the complex relationship between resource profiles and suicide attempts among emerging adults with disabilities. Another study limitation is the relatively small number of participants with disabilities who self‐reported information about suicide attempts, ranging from 1.8% to 2%, thereby posing challenges for statistical analysis and interpretation. To address this limitation, we aggregated data from two waves spanning early adulthood from 18 to 32 years. Similarly, there may be potential limitations in relation to self‐report questionnaires for youth with disabilities, particularly concerning the difficulty of understanding language and cognitive limitations. To address this issue, trained data collectors assisted adolescents who might have faced challenges in completing the survey independently during the administration of the Add Health survey. Although some of the standardized instruments used in the Add Health survey that are not specifically tailored for youth with disabilities its established reliability and validity in general adolescent populations, along with its brevity and ease of use, make them an appropriate use, including CES‐D‐9, in this study. Future research should expand the use of the instrument to include youth with disabilities, as well as specific disability types, to examine psychometric properties of the tools within these populations. In addition, we employed advanced statistical techniques like the Gaussian finite mixture model (GMM) to explore potential data patterns. Despite these efforts, the small sample size diminishes the statistical power of our analyses, precluding detection of meaningful associations or differences. Consequently, caution is warranted when interpreting the findings of this study. Future research with larger sample sizes or alternative methodologies may provide deeper insight into the relationship between disability and suicide attempts.

## Conclusion

This study is the first to identify distinct subgroups among youth with disabilities based on their resource profiles, including family socioeconomic status and social support, in relation to risk of suicide attempts. The study highlights the diversity of this population, shaped by their unique demographics, specific disability needs, familial and school contexts and social support systems. Some findings challenge traditional perspectives on suicide risk indicators by suggesting that structured mental health support and early intervention serve as protective factors. Future research should explore psychological management of youth with disabilities in relation to their specific needs to validate these findings and inform targeted suicide prevention strategies.

## Ethical considerations

This study complies with the Declaration of Helsinki, and all procedures involving human participants were conducted in accordance with institutional guidelines. The study protocol was reviewed and approved by the Institutional Review Board of Seoul National University (approval date: 6 October 2022; IRB no. 2210/004‐010). The data analysed in this study were obtained from the National Longitudinal Study of Adolescent Health (Add Health). In the original Add Health study, informed consent and/or assent (including parental consent where applicable) was obtained from all participants. Because the present study involved secondary analysis of de‐identified, nationally collected data, additional informed consent was not required.

**Key points**

**What's known?**

- Youth with disabilities face higher risks of mental health issues, including suicide attempts, during the transition to adulthood.
- Socioeconomic status and social support are key factors influencing mental health outcomes.

**What's new?**

- This study identified four resource profiles based on these factors, finding that youth in socioeconomically advantaged but socially isolated and socioeconomically disadvantaged but socially supportive groups had lower suicide attempt risks. Youth with learning disabilities showed reduced risk, possibly due to early psychological support.

**What's relevant?**

- Tailored mental health interventions and targeted screening for prior suicide attempts and suicide exposure could improve prevention strategies.

## Supporting information


**Appendix S1.** Identifying latent profiles of resource/support in youth with disabilities.
**Table S1.** Gaussian mixture models and fit indices.
**Figure S1.** Scaled mean of variables for the four‐class Gaussian mixture model when the number of group is 2, 3 or 5.

## Data Availability

The data that support the findings of this study are available on request from the corresponding author. The data are not publicly available due to privacy or ethical restrictions.
