# Supplementary material for: Resource profiles and suicide attempts in youth with disabilities
Source: J Child Psychol Psychiatry. 2026 Jan 30;67(6):976–87. doi: 10.1111/jcpp.70122 (PMC13170621; doi:10.1111/jcpp.70122)

**Resource Profiles and Suicide Attempts in Youth with Disabilities**

**Supporting Information**

**Appendix S1. Identifying latent profiles of resource/support in youth with disabilities**

To determine the optimal number of groups within laten populations based on financial stability, parent income, parent occupation, parent education, religiosity, social cohesion, family relation, peer relation, school attachment, and teacher relation, multiple models with varying numbers of groups were estimated to identify the model that best captures the underlying structure of the data. Fit indices, such as AIC, BIC, and ICL, for each group number were calculated, as displayed in Table S1. Based on AIC and BIC criteria, setting the number of latent groups to 6 significantly increased the model's complexity, hindering parsimony. Conversely, setting it to 2 groups, based on ICL criteria, failed to capture the complexity adequately. Thus, after considering both parsimony and complexity comprehensively, we determined that 4 groups were optimal. This setup has the lowest AIC and BIC among configurations with up to five groups, and the lowest ICL among configurations with three or more groups. Figure S1 visualizes the comparisons between the three models: 2, 3, and 5 groups.

**Table S1**

*Gaussian Mixture Models and Fit Indices*

| **Classes** | ***df*** | **LL^a^** | **AIC** | **BIC** | **ICL^b^** |
| --- | --- | --- | --- | --- | --- |
| Two-class | 131 | -44172 | 88606 | 89369 | -89381 |
| Three-class | 197 | -43695 | 87784 | 88932 | -89077 |
| Four-class | 263 | -43445 | 87416 | 88948 | -89128 |
| Five-class | 329 | -42424 | 85506 | 89442 | -87479 |
| Six-class | 395 | -41365 | 83520 | 85820 | -85971 |

^a^ Log Likelihood; ^b^Integrated complete-data likelihood

**Figure S1**

*Scaled Mean of Variables for the Four-Class Gaussian Mixture Model when the Number of Group is 2, 3, or 5*


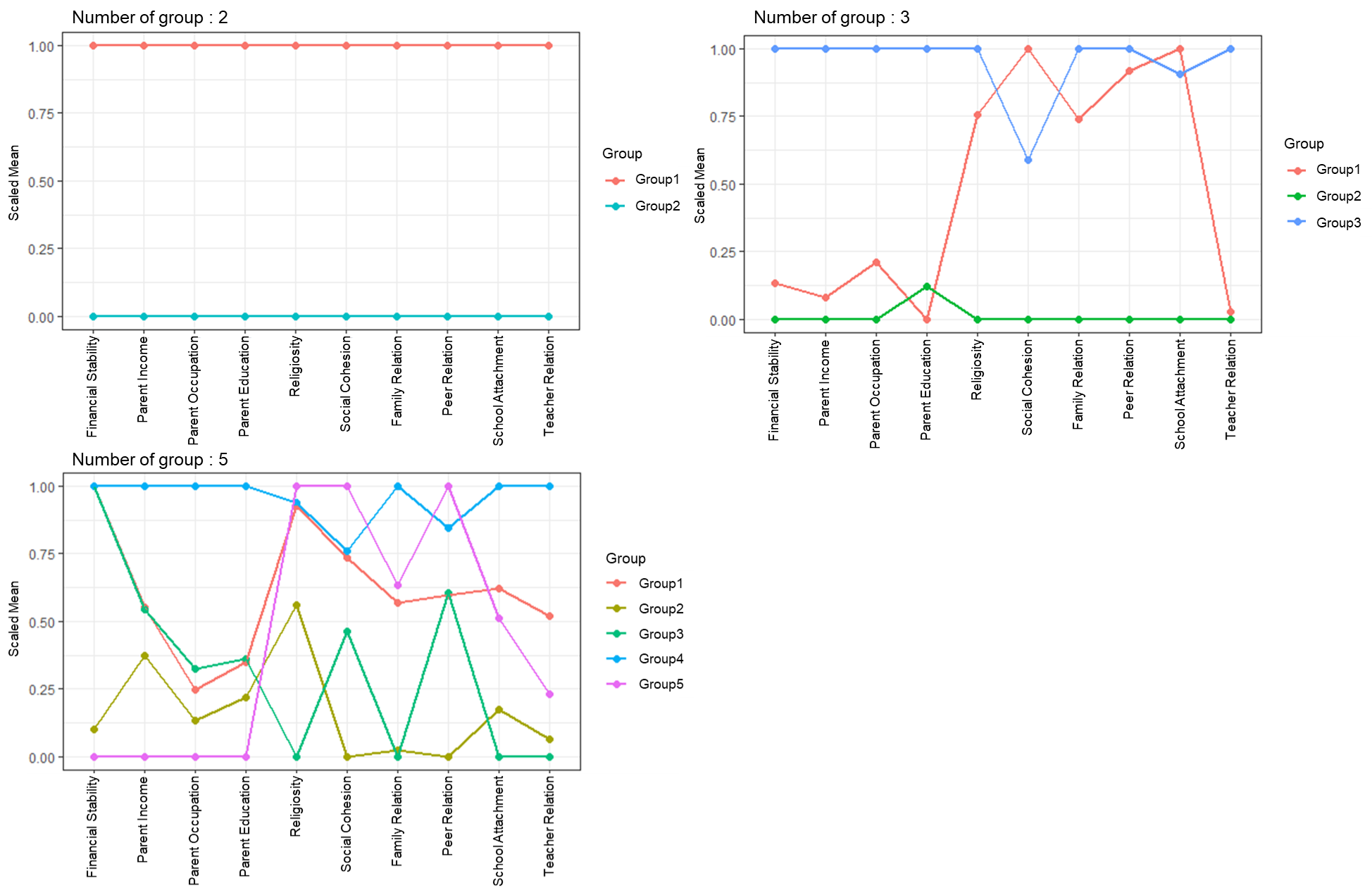

Supplement: Supplementary file 1 — Appendix S1. Identifying latent profiles of resource/support in youth with disabilities. Table S1. Gaussian mixture models and fit indices. Figure S1. Scaled mean of variables for the four‐class Gaussian mixture model when the number of group is 2, 3 or 5. [file JCPP-67-976-s001.docx]
